# The prevailing O serogroups among the serologically differentiated clinical *Proteus* spp. strains in central Poland

**DOI:** 10.1038/s41598-021-98228-w

**Published:** 2021-09-23

**Authors:** Dominika Drzewiecka, Agata Palusiak, Małgorzata Siwińska, Agnieszka Zabłotni

**Affiliations:** Department of Biology of Bacteria, Faculty of Biology and Environmental Protection, University of Lodz, Banacha 12/16, 90-237 Łódź, Poland

**Keywords:** Bacterial pathogenesis, Infectious-disease epidemiology

## Abstract

In the years 2006–2011, 617 *Proteus* spp. strains isolated mostly from urine and wounds or other clinical sources were collected in Łódź, Poland, to determine the offensive O serotypes frequently occurring among patients. *P. mirabilis* exhibited the most intensive swarming growth and was dominating species (86.9%), followed by *P.* genomospecies, *P. vulgaris*, and *P. penneri*. Ninety four per cent strains were recognized as S (smooth) forms. Serological studies (involving ELISA—enzyme-linked immunosorbent assay and Western blotting using native and adsorbed rabbit antisera) enabled classification of 80% S isolates into respective *Proteus* O serogroups among the 83 ones, described so far. The remaining strains seemed to be serologically unique. Despite the observed big serological variety of *Proteus* spp. isolates, we found the O78 serogroup recently described in Poland as dominating and identified other widespread serotypes: O3, O6, O10, O11, O27, O28, and O30 reported earlier as predominating also in other countries; O77 and O79 detected lately in Poland; O16, O18, O20, and O50. No unique structural feature of the prevalent O serotypes has been indicated. However, the prevalence of some O serogroups indicates that particular serotypes may be in some ways beneficial to the strains producing these kinds of O antigen.

## Introduction

*Proteus* spp. bacteria are peritrichously flagellated members of *Morganellaceae* family distinguished from the other representatives of the order *Enterobacterales* by intensive and spectacular swarming growth on solid media as a result of the multicellular differentiation phenomenon^1,2^. These bacilli are detected in natural environments (water or soil) and in many species of wild and domestic animals. They have also been found to be a component of natural faecal microbiota in a part of human population. On the other hand, *Proteus* spp. are opportunistic pathogens, which may affect mainly immunocompromised individuals and cause infections of the urinary tract and wounds, bacteraemia, abscesses in many organs or other infections^3^. Among the pathogenic species, *P. mirabilis* is most common followed by less frequently isolated *P. vulgaris*, *P. penneri*, *P. hauseri*, and three genomospecies without names (*P*. genomospecies 4, 5, and 6). Lack of biochemical reactions which would reliably distinguish between different *P.* genomospecies resulted in limited knowledge on their characteristics and isolation frequency^4,5^, when compared to well-described *P. mirabilis*. Urinary tract infections (UTIs) of *P. mirabilis* aetiology are frequently catheter-associated—CAUTIs, recurrent and dangerous due to the frequent and severe complications like *pyelonephritis* and *urolithiasis*^2^.

Among numerous virulence factors of *Proteus* bacteria, the lipopolysaccharide (LPS) should be emphasized as an endotoxin and an important antigen determining the serological specificity of *Proteus* spp. strains. The LPS core region in the genus *Proteus* is structurally and serologically diverse, which has led to the formation of the R (rough) typing *Proteus* scheme containing 11 R serotypes^6^. However, mainly the highly immunogenic O-polysaccharide region determines the serological specificity of LPS S (smooth) forms, which has become a basis for the classification of *Proteus* strains into 83 O serogroups^7,8^. The first O type scheme for *P. mirabilis* and *P. vulgaris* strains including 49 O serogroups was founded by Kauffmann^9^. Later, Larsson^10^ indicated O3, O6, O10, O11, O13, O23, O24, O26, O27, O28, O29, and O30 as the most prevalent serogroups in many countries. The Kauffmann’s scheme was further extended with other O serogroups containing all *Proteus* species^7^ but only a few *P. penneri* and *P. mirabilis* strains come from Poland^11,12^. Gathering a new wide collection of more than 600 *Proteus* spp. clinical isolates from the Łódź region (central Poland) enabled us to study their serological differentiation. Up to date, serologically unique strains from the collection have formed six new serogroups O77-O82^13–18^ and two new subgroups in O8 and O11 serogroups^19,20^. In this work the serological variety of the whole collection of *Proteus* spp. strains is shown focusing on *Proteus* O serogroups most widespread in central Poland (Łódź region).

## Methods

### Bacterial strains, physiological tests, LPSs

**Figure 1:**
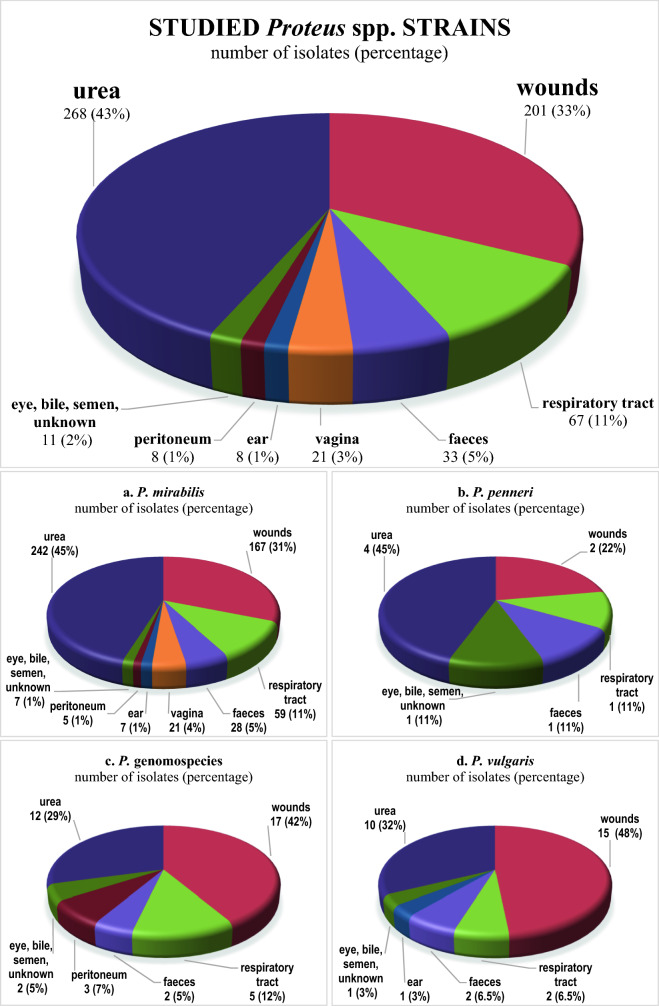
The sources of isolation of the whole collection of 617 *Proteus* spp. strains and separately: *P. mirabilis* isolates (**a**), *P. penneri* isolates (**b**), *P.* genomospecies isolates (**c**), and *P. vulgaris* isolates (**d**) [this work and^13–20^].

Six hundred and seventeen *Proteus* spp. strains (including the 24 strains reported earlier^13–20^) were isolated from different clinical sources (Fig. 1) in the Łódź city, central Poland, in that number 606 ones from infected individuals. The bacteria were also detected in faeces from eleven carriers out of 189 individuals with no problems in the digestive tract. The strains were kindly gifted in 2006–2011 by the four biggest medical laboratories in Łódź (Poland): laboratories in Barlicki Hospital and in Biegański Hospital, as well as „Synevo” laboratory and „Diagnostyka” laboratory. The strains were isolated both in hospitals and from non-hospitalized patients, coming from Łódź city and Łódź region. We identified all the isolates’ species by the cultivation on the media proposed by Senior^21^, modified as described^13^ and used for testing ornithine decarboxylation, phenylalanine deaminase and urease production, mannose fermentation and indole formation. Additional tests (for L-rhamnose fermentation, lipase production, Jordan’s tartrate and DNase) were applied for the strains recognized as *P.* genomospecies, as described^4,15^ (Table 1). All strains were retained in stocks (Luria Broth (LB) cultures with 25% glycerol) at − 80 °C.

**Table 1:** Biochemical features and frequency of *Proteus* species occurrence (including the isolates reported earlier^13–20^).

	*Proteus* species					
	*P. m.*	*P. v.*	*P. p.*	*P. h.*	*P.* gsp. 4	*P.* gsp. 5/6
Metabolic feature						
Phenylalanine deaminase	+	+	+	+	+	+
Mannose fermentation	−	−	−	−	−	−
Urease production	+	+	+	+	+	+
Indole production	−	+	−	+	+	+
Ornithine decarboxylation	+	−	−	−	−	−
Salicin fermentation	−	+	−	−	−	−
Esculin hydrolysis	ns	+	ns	−	−	−
Rhamnose fermentation	ns	ns	ns	−	+	−
Lipase production	ns	ns	ns	−	+	+
DNase production	ns	ns	ns	−	+	+
Frequency						
Strains no	536	31	9	0	9	32
Percentage	86.87%	5.02%	1.46%	0%	1.46%	5.19%

ns, not studied.

*P.m., Proteus mirabilis.*

*P.v., Proteus vulgaris.*

*P.p., Proteus penneri.*

*P.h., Proteus hauseri.*

*P.*gsp., *Proteus* genomospecies.

S-R tests included both the thermal stability in the boiled broth medium test and the pseudoagglutination test^22^. Additionally, the swarming growth ability on Luria Broth medium with 1.5% agar was studied, according to Wilkerson and Niederhoffer^23^. In these tests, *P. penneri* 13 rough strain coming from the collection of the Laboratory of General Microbiology, University of Łódź, was applied as a control.

Thermal stability of broth cultures: 18-h bacterial cultures in LB liquid medium were heated (2.5 h at 100 °C). Bacterial R forms precipitated from the broth and formed a sediment (the broth above it was clear), which differentiated them from S forms (not precipitating, stable suspended in the whole broth volume). In contrast, SR phenotypes formed a small amount of sediment but the broth above it remained cloudy.

Pseudoagglutination assay: the bacterial cells from 18-h nutrient broth agar slant cultures were suspended in a drop of 0.85% NaCl in a microscopic slide. Self-agglutination was seen for R forms of bacteria while S forms did not agglutinate.

Swarming growth ability: plates with the LB medium supplemented with agar (1.5%) were dried to remove drops of water from their surface. LB liquid culture of each strain was spot inoculated (10 μl) in the center of an LB plate and incubated for 24 h at 37 °C. Over this period concentric swarm zones were counted and a radius of the total swarming growth area was measured.

Biomasses of the studied strains were obtained by 18-h cultivation on a nutrient broth medium supplemented with 0.2% glucose, with aeration, at 37 °C. The strains were killed by 1% phenol, centrifuged, washed with distilled water and stored in + 4 °C (wet biomass) or lyophilized (dry biomass).

LPSs were extracted from strains’ dry biomass in 45% phenol for 5 min at 65 °C using the classic phenol-water method, published by Westphal and Jann^24^. After centrifugation (4845×*g*) the water phase containing LPS with long OPS chains was collected and the extraction was repeated. The water phases were combined, dialyzed from phenol for 48 h and centrifuged (4845×*g*) to remove the cell debris. The LPS solution was adjusted to 2% CH_3_COONa and a crude LPS was precipitated by the addition of 2 v of 96% ethanol. The LPS precipitate was dissolved in distilled water, dialyzed from the salt and ethanol remains (24 h, 4 °C) and lyophilized.

LPSs and biomasses used as the antigens in SDS-PAGE were suspended in loading buffer, boiled for 10 min. and then treated with proteinase K (10 mg/ml) for 1 h at 60 °C^15^.

### Serological investigation

We applied ELISA (enzyme-linked immunosorbent assay) and Western-blotting methods using bacterial biomasses and LPS preparations (extracted from selected studied strains or coming from our collection) as a source of O antigens as well as polyclonal rabbit sera specific to all the O serotypes reported in the genus *Proteus* (from our collection), as described earlier^15,25^.

Additionally, to confirm the classification of studied strains to 14 most numerous serogroups, we used the respective 14 antisera adsorbed by wet bacterial mass of cross-reacting strains, previously washed in PBS. The biomass was suspended in a serum diluted (1:50) in PBS in a proportion of 1:10 and incubated for 30 min. on ice. The cells were removed from the serum by centrifugation and the process was repeated twice^13^. Then, in ELISA, a lack of the reaction of the adsorbed antiserum with the homologous LPS confirmed the identity of the cross-reacting O antigen with the reference LPS due to removal of all the specific antibodies during adsorption. The stronger reaction observed in ELISA or Western blotting after adsorption, the lower similarity was proved.

In ELISA, LPSs (50 ng per well) or the bacterial biomasses (5 µg per well) were applied for coating the F96 Maxisorp Nunc-Immuno plates (12 h, + 4 °C). After washing the plates twice with distilled water to remove the residual antigens, the uncoated sites in the wells were blocked with casein buffer (2.5% casein, 240 mM of NaOH, 1.5 mM of KH_2_PO_4_, 8 mM of Na_2_HPO_4_, pH 7.2) for 1 h at 37 °C, to avoid subsequent unspecific adsorption of antibodies. The plates were doubly washed with phosphate buffer saline (PBS, 136.8 mM of NaCl, 2.7 mM of KCl, 8 mM of Na_2_HPO_4_, pH 7.2) and incubated with the appropriate polyclonal rabbit antiserum (twofold serially diluted in 50 µl of casein buffer) for 1 h at 37 °C. After triple washing the plates with PBS (to remove the unbound immunoglobulins), the rabbit-IgG specific goat antibodies labelled with horseradish peroxidase (Jackson ImmunoResearch) diluted 1:5.000 in casein buffer were added (50 µl/well) and the plates were incubated as described above. Triple washing with PBS and a single one with substrate buffer (0,1 M of C_6_H_5_Na_3_O_7_, pH 4.5) were applied and the solution of 2,2’-azino-bis(3-ethylbenzothiazoline-6-sulfonic acid) diammonium salt (ABTS) in substrate buffer with the addition of 3% H_2_O_2_ (1:25) was used (50 µl/well) as a substrate for peroxidase. After 30 min. of incubation at 37 °C, the colour reaction was stopped by the use of oxalic acid (222 mM). Antibody titers were determined, in relation to a blind sample (ABTS solution), by measuring the absorbance (A_405_) using Multiskan Go microplate reader (Thermo Scientific). Absorbance ≥ 0.2 was considered as indicating a positive reaction.

In Western blotting, bacterial biomass or LPS in the loading buffer (5–8 µg per lane of polyacrylamide 3% stacking gel) were separated during sodium dodecyl sulphate polyacrylamide gel electrophoresis (SDS PAGE) on 200 V. Then, the samples were transferred to nitrocellulose (Whatman Schleicher & Schuel) in transfer buffer (25 mM Tris/HCl, 192 mM of glycine, 20% methanol) on 100 V for 1 h. The membrane was fixed for 5 min. with the solution of 10% isopropanol and 10% acetic acid in water and blocked with 10% skimmed milk in dot-blot buffer (50 mM of Tris/HCl pH 7,4, 200 mM of NaCl) for 1 h. Then, the nitrocellulose was incubated with appropriate rabbit antiserum diluted 1:500 in dot-blot-10% skimmed milk buffer (2 h) and, after washing with the dot-blot buffer, with the rabbit-IgG specific goat antibodies conjugated with alkaline phosphatase (Jackson ImmunoResearch) diluted 1:5000 in the dot-blot-10% skimmed milk buffer (2 h). AP Conjugate Substrate Kit (Bio-Rad) was applied as a substrate of alkaline phosphatase to obtain a colour reaction, which was stopped by washing the membrane in distilled water. Western blotting reactions were scanned by means of Canon Toolbox 4.9.

## Results

### Physiological features

Among the 617 studied strains, urine isolates and wound isolates (including bacteria from pus, bedsores, fistulas, skin and wound swabs, and abscesses) were dominating. Other frequent sources of isolation were connected with the respiratory tract (bronchoaspirates, sputum, and the pharyngonasal cavity), vagina, and faeces, while strains from other sources were isolated rarely (Fig. 1). It was also shown that the eleven *Proteus* spp. strains (nine *P. mirabilis*, one *P. vulgaris*, and one *P.* genomospecies 5/6 including four strains reported earlier^13,14,20^) were inhabiting the intestines of 5.8% from 189 individuals (carriers).

The strains species were identified on the basis of the metabolic features of the isolates (Table 1). *P. mirabilis* species was definitely predominant, while the contribution of the other detected *Proteus* species was much lower (Table 1). Totally, 41 isolates (6.6%) were found to belong to *P.* genomospecies 4 or 5/6, the two last are still impossible to distinguish on the grounds of their metabolic properties^4,5^, while no isolate was classified to *P. hauseri* species. Thus, it can be assumed that *P.* genomospecies are isolated from clinical sources with a frequency comparable to that observed for *P. vulgaris* and all the isolates belonging to the so called *P. vulgaris* group together account for 11.7% of the whole collection, where *P.* genomospecies 4 is the rarest. Contrary to *P. mirabilis* and *P. penneri* (Fig. 1a,b), among *P. vulgaris* and *P.* genomospecies the isolates from wounds were found to dominate, while the isolates from urine were less common (Fig. 1c,d).

To select the isolates which can be typed to O serogroups (S forms), we conducted S-R tests. Thirty seven studied strains (6% of the collection) which were totally or partly non-thermal stable and agglutinated in 0.85% NaCl were recovered as R or S/R forms possessing LPS deficient in O polysaccharide or LPS with only one O-repeating unit, respectively. R forms constituted only 4.9% of *P. mirabilis* strains (26 out of all collected *P. mirabilis* isolates), while in *P. vulgaris* it was 9.7% (three out of 31 *P. vulgaris* isolates), in *P.* genomospecies 4 and 5/6—14.6% (six out of 41 isolates), and in *P. penneri*—22.2% (two out of nine isolates). As the shortened LPS without O-polysaccharide part does not guarantee a proper and stable truncation of the flagella, the swarming growth of R forms is usually decreased or stopped. As expected, the majority of R strains were not able to swarm on the surface of solid Luria Broth medium. However, among the smooth strains, the non-swarming ones were also detected. Most of *P. mirabilis* isolates (more than 90%) were found to be able to swarm and 70% exhibited intensive swarming growth (on the distance > 30 mm within 24 h). In the case of *P. penneri*, non-swarming strains constituted 22% (R forms only) while the other isolates were able to swarm effectively. The weakest swarming growth ability was observed among *P. vulgaris* and *P.* genomospecies isolates as 50% of strains have not showed this ability or could swarm very weakly (< 10 mm within 24 h).

### Serological features

A total of the 580 S strains (possessing an O antigen in the LPS molecules) were serologically investigated to classify them into proper O serogroups in the genus *Proteus*. The results for 24 of these strains classified to O2, O8, O11, O77—O82 serogroups, respectively, had been reported previously^13–20^ .

Biomasses of the S strains were studied in ELISA with rabbit polyclonal antisera specific to all the *Proteus* O serogroups described so far. Strong cross reactions comparable to the ones in the homologous systems (mostly to the titre 1:128000 or more) were the first indication on the serological type of the studied strains. But the obtained results were not always so clear.

Several biomasses were not recognized by any antisera (the O antigens present on the cells were possible to be masked by other cell wall components) and some cross reacted with more than one antiserum achieving various titres (other surface antigens may have also been recognized by the antibodies present in the used rabbit antisera). In these cases, LPS preparations were extracted from the biomasses by the phenol-water procedure^24^. Purified LPS antigens coated on the ELISA plates might be better exposed and better accessible to specific antibodies. Indeed, in consequence the O serotype of several such strains was identified.

These results allowed recognizing the similarity of most of the isolates (447/556 S forms studied in this work) to particular O serotypes among 83 *Proteus* O serogroups recognized so far, while the remaining strains seemed to be serologically unique and were considered to represent new O serogroups in the genus *Proteus*. One hundred and fifty six isolates had been preliminarily classified into 47 O serogroups (data not shown) formed by fewer than 10 studied strains. More than a half (291) of the studied here 556 S *Proteus* spp. clinical strains were initially considered as belonging to 14 predominating O serogroups consisting of 10 or more strains: O3, O6, O10, O11, O16, O18, O20, O27, O28, O30, O50, O60, O77, and O78.

To confirm that consideration, the sera specific to the 14 most prevalent O serotypes were adsorbed by the cross-reacting biomasses and their reactions with the homologous LPSs were analyzed in ELISA. In most cases, the reactions of the antisera in the homologous systems were totally abolished due to the removal of all LPS-specific antibodies by the cross-reacting biomasses, indicating their antigenic identity and confirming the initial serological classification (Table 2). However, in some cases the adsorption was not complete and some remaining fractions of antibodies still reacted with homologous LPSs, although the reactions were obviously weaker than the ones of the native antisera (Table 2). In these 34 cases, some differences in the strains serospecificity were responsible for not full adsorption of the antisera. The antibodies which could not be removed from the adsorbed antisera should be specific to some epitopes, which are present in the reference homologous LPSs (core region or O antigen) but absent in the cross-reacting studied strains. Thus, such reactions indicated more or less antigenic similarity but not the identity of the studied strains and the reference LPSs.

**Table 2:** Cross reactions of studied strains with the respective O antisera (native and adsorbed by the studied strains’ biomass) in ELISA.

O antiserum	Reciprocal titre of antiserum reacting with		
	Homologous LPS	Studied strains biomass	Homologous LPS after adsorption (number of adsorbing strains)
O3	256,000	32,000–512,000	< 1000 (17) 2000–8000 (4)
O6	64/128,000	32,000–128,000	< 1000 (19)
O10	512,000	64,000–512,000	< 1000 (9) 4000–32,000 (12)
O11	256,000	64,000–512,000	< 1000 (31) 2000–4,000 (2)
O16	256,000	32000–512,000	< 1000 (22) 16,000–64,000 (3)
O18	128,000	64,000–512,000	< 1000 (11)
O20	512,000	256,000–2,048,000	< 1000 (24)
O27	1,024,000	64,000–2,048.000	< 1000 (18)
O28	64/128000	32000–512000	< 1000 (13) 4,000 (2)
O30	5,12000	128000–1,024,000	< 1000 (12)
O50	64/128,000	32,000–512,000	< 1000 (11) 4,000 (1)
O60	512,000	64,000–512,000	< 1000–1000 (10)
O77	256,000	64,000–512,000	< 1000 (16)
O78	512/1,024,000	32,000–512,000	< 1000 (44) 2000–32,000 (10)

**Figure 2:**
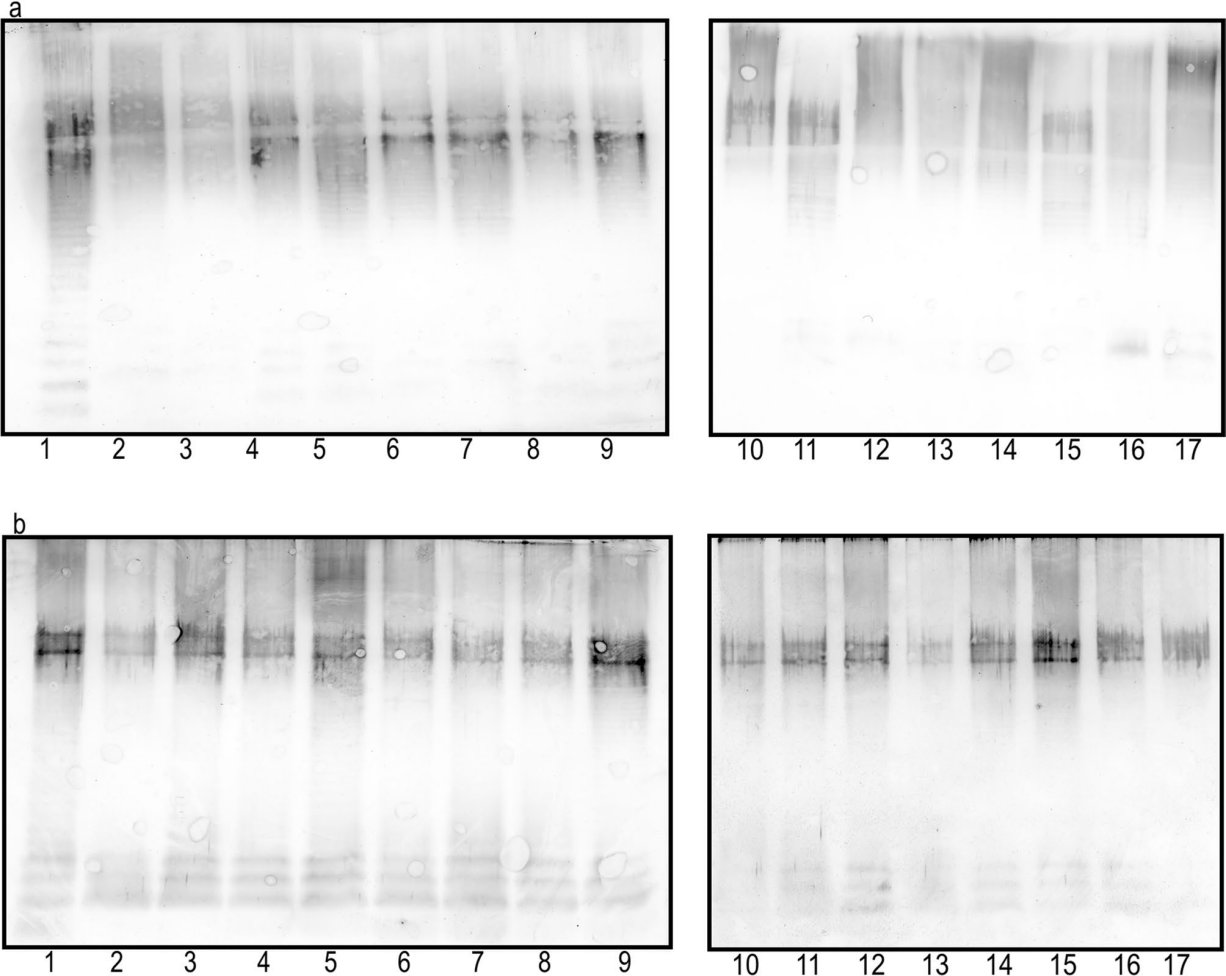
Western blots of O77 LPS (1) and proteinase K-treated biomasses (**a**) or LPSs (**b**) of the strains (2–17) classified to O77 serogroup with O77-specific serum.

**Figure 3:**
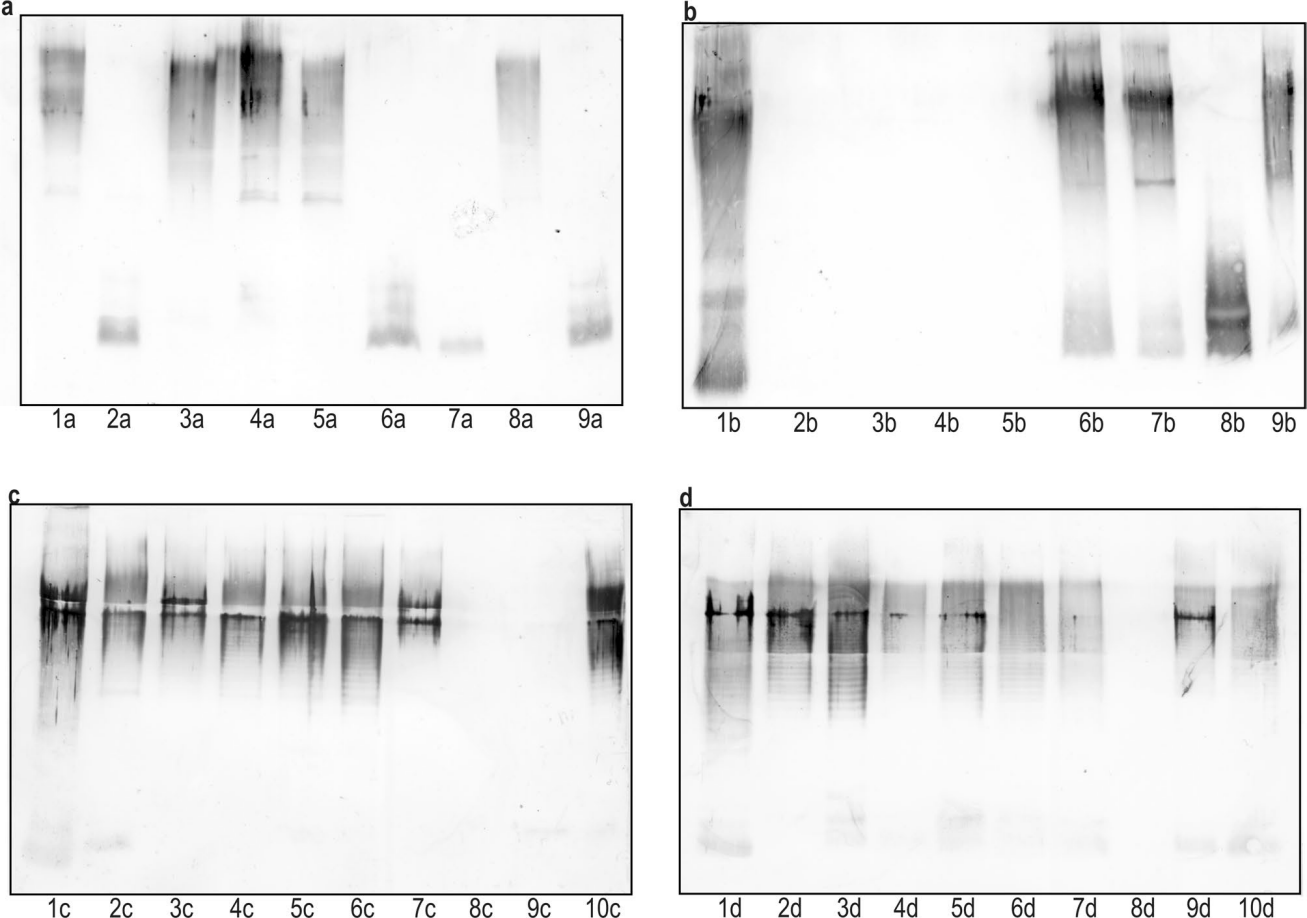
Western blots of homologous LPSs (1) and proteinase K-treated biomasses of selected strains (2–10) preliminarily classified to O3 serogroup (**a**), O10 serogroup (**b**), O28 serogroup (**c**), and O50 serogroup (**d**) with the respective antisera.

**Figure 4:**
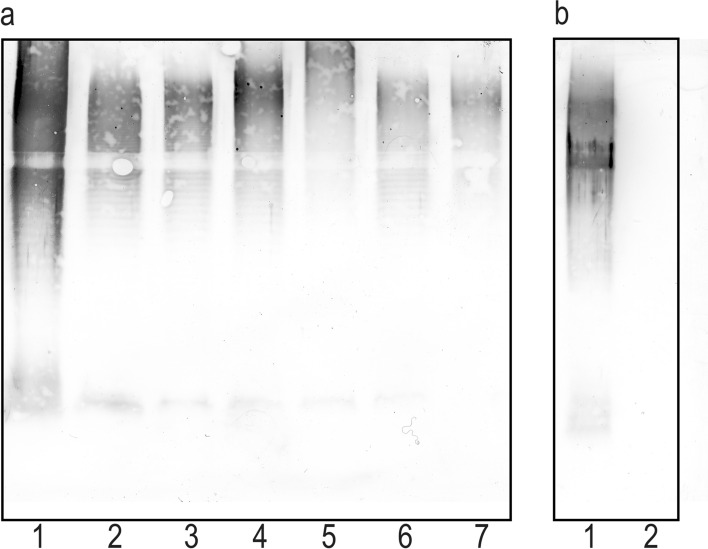
Western blots of O78 LPS (1) and proteinase K-treated biomasses of selected strains (2–6) classified into O78 serogroup with O78 antiserum (**a**) and O78 antiserum adsorbed by the strain 2 (**b**).

The nature of the cross reactions, observed in ELISA, was visualized in the Western-blotting method using LPS preparations obtained by proteinase-K treated biomasses as antigens (Figs. 2, 3, 4). The reactions are similar and comparable to the ones observed for the LPS preparations obtained using phenol-water extraction, which is demonstrated on the example of strains classified to O77 serogroup (Fig. 2a,b). By the degradation of proteins, we could remove possible non-specific cross reactions caused by protein antigens rather than by O antigens^26^. Indeed, the initially observed in ELISA reactions of several biomasses not fully adsorbing the respective antisera, could have been caused by protein antigens, as they were not confirmed in Western blots. That was the case of four strains (Fig. 3b, paths 2–5) preliminarily classified into O10 serogroup, two strains (Fig. 3c, paths 8, 9) classified into O28 serogroup, and one strain (Fig. 3d, path 8) classified into O50 serogroup, which displayed completely no reaction after proteinase-K treatment in Western blots.

The Western-blotting technique also allowed localizing LPS epitopes responsible for the strong cross reactions initially observed in ELISA. The epitopes may have been present in the O-specific part and/or in the core region of the LPSs exposed on the biomasses of the studied strains. Indeed, among the strains preliminarily classified into O10 serogroup on the basis of strong reactions of their biomasses in ELISA, one isolate showed in Western blot reactions only in the fast-migrating fractions corresponding to the lipid A-core region, while there were no reactions observed for the slowly migrating LPS bands additionally possessing O-polysaccharide chains (Fig. 3b, path 8). Similar results were obtained in the case of as many as four strains (Fig. 3a, paths 2, 6, 7, and 9) initially classified into O3 serogroup. The O3 and O10 antisera are visibly rich in antibodies recognizing core-localized common epitopes and responsible for strong cross reactions observed both in ELISA and in Western blots. Obviously, these five strains cannot be included in O3 or O10 serogroups, although they may form R serogroups.

Noteworthy, the 15 strains initially classified into the most numerous O78, O16, and O11 serogroups but not fully adsorbing the respective O78, O16, or O11 antisera (Table 2) displayed in Western blots visible strong reactions of the slowly-migrating LPS molecules possessing O polysaccharides (O-PS), which is showed on the example of the strains (2–7) classified to O78 serogroup (Fig. 4a, paths 2–7). The use of the O78, O16, or O11 antisera adsorbed by the respective biomasses in the Western-blotting technique proved that the observed serological differences refer mainly to the O-specific parts of these strains’ LPS. As the antisera contain small amounts of anti-core antibodies, the possible differences in core antigens were hard to be detected. The adsorbed O78, O16, and O11 antisera did not react with the respective adsorbing biomasses, which confirmed the properly conducted adsorption processes (control), but still in these three antisera there were remaining some fractions of antibodies recognizing the epitopes located in the homologous O antigens (slowly-migrating LPS bands) but absent in the studied strains. The Western-blotting results for these 15 strains were similar and they are shown on the example of O78 antiserum adsorbed with strain 2 (Fig. 4b). Thus, the 15 strains were recognized as representing different subgroups within O78, O16, or O11 serogroups, respectively.

## Discussion

In the years 2006–2011 a total of 617 *Proteus* spp. strains were collected in Łódź, Poland (Fig. 1) including the 24 clinical strains reported earlier^13–20^. What is worth noticing, no strains were collected from blood, although *Proteus* spp. bacteria may also cause severe bacteremia and sepsis^2,27^. Urine and wound isolates were prevailing (Fig. 1). Frequent isolation of *P. mirabilis* (Table 1) is not surprising, as the species is regarded as the most virulent and widespread species in the genus *Proteus*, especially in patients suffering from nosocomial urinary tract and wound infections and it is still the most frequently isolated species among *Proteus* spp.^2^. *P.* genomospecies 4, 5, 6, or *P. hauseri* are not usually distinguished from *P. vulgaris* in the routine laboratory work and so there are very few reports available on their occurrence in patients. Similarly to our results, O’Hara et al.^4^ and Janda et al.^5^ studying several *P. hauseri*, *P.* genomospecies 4, 5, and 6 strains, mostly from human sources, found *P.* genomospecies 5 and 6 to be the most numerous. Our results confirm the low frequency of *P. penneri* isolation from clinical sources^28,29^*.* Recently, Dai et al*.*^30^ have proposed a new molecular MLSA (multilocus sequence analysis) technique for identification of *Proteus* species, suggesting a reclassification of *P. cibarius* and *P.* genomospecies 5 into the formerly described species *P. terrae* isolated from soil. Among 210 isolates from clinical specimens and food in China, the authors found *P. terrae* as being the second most abundant species after *P. mirabilis*, when *P. vulgaris* and *P. penneri* as the third and fourth ones, respectively.

R forms which are regarded as less virulent, we found among the isolates belonging to all the identified *Proteus* species. However, in the most pathogenic *P. mirabilis* species, the smallest number of R forms was detected and the best swarming ability was noticed which is considered to be an important virulence factor. *P.* genomospecies and *P. vulgaris* isolates demonstrated weaker swarming growth in comparison to *P. penneri* strains, similarly to the results achieved by Kwil et al.^31^.

Serotyping is an important way of typing and classification of bacteria, especially in diagnostics and epidemiology of some pathogenic genera like *Salmonella*. In the case of this genus, besides the classical phenotypic methods new precise molecular tools are recommended, such as whole genome sequencing (WGS)-based serotype prediction and microarray-based method^32^. WGS was also applied in studies of a *P. mirabilis* strain virulence^33^. Many O-antigen gene clusters were analyzed and O serotype-specific suspension array for detecting selected *Proteus* serotypes has been proposed^34^. However, the prevalence and significance of particular serogroups in patients have still not been obvious.

**Figure 5:**
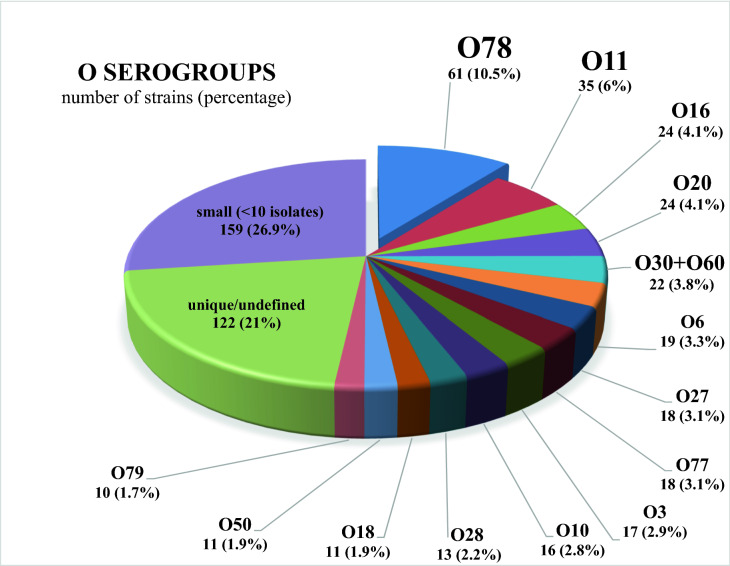
Classification of 580 S *Proteus* spp. strains into O serogroups [this work and^13–20^].

Serological studies of Polish strains confirmed that the studied strains possess various core types which is typical in the genus *Proteus*. Frequently, strains forming a common R serogroup do not belong to a common O serogroup and the strains classified into one O serogroup may differ in their core-region serospecificity^6,11^. Our studies showed a big serological diversity among *Proteus* spp. strains isolated recently from patients in central Poland. However, half of the isolates (299) including 18 strains reported before^13–15,19^ belong to 15 most numerous O serogroups, comprising ten or more studied strains (Fig. 5). O78 serogroup is the biggest one, formed by 61 studied strains (10.5% of 580 collected S strains). This serogroup together with O77 and O79 serogroups were described as new ones not long ago by Drzewiecka et al.^13,14^ and Arbatsky et al.^15^ and they currently include only the isolates from Poland. O50 serogroup, in turn, was reported in 2003^35^ and has so far been represented by *P. mirabilis* strain TG 332 described earlier as being serologically unique^36^. O60 serogroup was also formed in 2003^37^ for a non-clinical *P. myxofaciens* isolate from a gypsy larva. Lately, the species has been proposed to be excluded from the genus *Proteus*^38^. However, our results clearly indicate that O60 serotype exists among *P. mirabilis* and *P.* genomospecies clinical strains. The other 10 most prevalent serogroups have been included in the first classic serological scheme proposed for *P. mirabilis* and *P. vulgaris* bacteria^9^. What is interesting, the majority of these predominant serogroups had been previously reported as frequently found among hundreds of clinical *P. mirabilis* and *P. vulgaris* isolates from urine, faeces, blood, or unknown sources. Summarizing these data, Larsson^10^ indicated O3 serogroup as dominating in all reports and O10, O13, O26, O28, and O30 serogroups as the most prevalent ones. O6, O11, O23, O24, O27, and O29 serogroups were also widely distributed. Analyzing the serological properties of 99 Swedish and 24 Polish *P. mirabilis* strains from urinary tract infections, Kaca et al.^12^ also found O10 and O30 to be the most numerous serogroups. However, the authors applied only 20 *Proteus* O-specific sera in their studies so the attachment of the strains to the other prevalent serogroups (*e.g.* O6, O26, O28, or O29) was not analyzed. In our studies, some of the prevalent serogroups turned out to be multi-species (Table 3), including not only the ubiquitous *P. mirabilis* but also the other species. Three *P. penneri* isolates were classified into O10 serogroup (so far found mostly in O17, O61, O64, and O65 serogroups^11,39^ including also *P. mirabilis* and/or *P. vulgaris* strains^7^), several *P.* genomospecies strains were classified into O11, O60, O78, and O79 serogroups and two *P. vulgaris* isolates into O27 and O78 serogroups. In all the prevalent serogroups, the isolates from urine and/or wounds were most numerous and we could not see any relations between serotypes and sources of isolation.

**Table 3:**
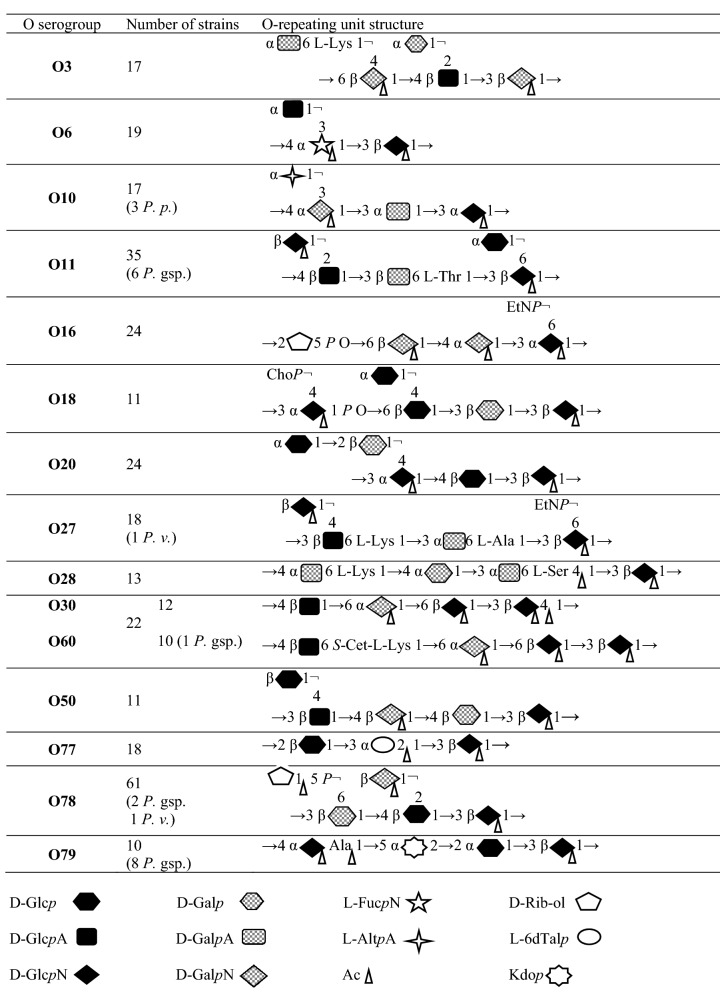
The O repeating unit chemical structures^7,15^ of the most prevalent *Proteus* spp. O serotypes in central Poland. The total number of strains includes non-*P. mirabilis* strains number (in parenthesis). Sugar components and acetyl groups are imagined as symbols:

O78, O16, and O11 serogroups seem to be heterogenous. Further chemical analysis may show the differences in the structures of O antigens, responsible for their slight serological variety observed within these serogroups and will allow the formation of new subserogroups or new cross-reacting O serogroups.

It can be seen that for many years, *Proteus* spp. strains belonging to O3, O6, O10, O11, O27, O28, and O30 serogroups have been most frequently isolated in many different countries. What is worth noticing, the *Proteus* O60 antigen structure resembles that of the *Proteus* O30 antigen^7^ so before the O60 serogroup was created in 2003^37^, O60 isolates could be classified to O30 serogroup due to their mutual similarity and strong cross reactivity revealed in our work (data not shown). It should be noted that at present, O78 serogroup obviously seems to be the most prevalent among Polish patients from the Łódź region accounting for 10.5% of all S isolates, although its predominance is not big. The prevalence of the serotypes dominating over others may be connected with some features of their O antigens. However, it is difficult to indicate common structural O-PS features (Table 3) distinguishing the biggest serogroups from the smaller ones, *e.g.* the compounds present exclusively in the frequent O antigens and, simultaneously, absent in the rarely found O serotypes^7^. Among the 15 most numerous serotypes, both branched and linear O-PS repeating units can be found, which are built of three-to-five sugars, including glucose, galactose, and their derivatives frequently occurring in *Proteus* O-PSs. For example, the repeating unit of the most ubiquitous O78 antigen is composed of five different sugars and sugar derivatives. Three of them constitute the main chain and two form side branches. This antigen is not characterized by any unusual or unique structural feature. It shares disaccharide fragments with several *Proteus* O antigens, but no cross reactions have been noticed^14^. It is possible that the most prevalent *Proteus* O serotypes may be especially connected with some virulence factors, like it is observed e.g. in *Vibrio cholerae* O1^40^ or with drug resistance, which is the case of *P. mirabilis* O78 (unpublished data).

## Conclusion

In general, clinical *Proteus* spp. isolates from central Poland are characterized by high serological diversity and include the representatives of almost all described O serogroups. These results may suggest that either *Proteus* spp. strains do not intensively transmit from one patient to another or they are able to rapidly change their antigenic specificity by avoiding the immune response from host organisms, increasing their own virulence and adapting to environmental conditions. Such examples of antigenic variation are known in other Gram-negative genera and may be caused either by mutations in O antigen genes or by inter/intra-species lateral transfer of O antigen genes by homologous recombination, IS elements, plasmids, or bacteriophages^41^.

The genus *Proteus* is currently divided into 83 O serogroups^8^ and their number continually grows exceeding numbers of serogroups in many other Gram-negative bacteria like the genera: *Shigella* or *Salmonella* (each 46 O serogroups), *Yersinia* (~ 35 O serogroups), *Citrobacter* (43 O serogroups), *Pseudomonas* (~ 30 O serogroups), and others, except for *Escherichia coli* (~ 180 O serogroups) and *Vibrio cholerae* (~ 200 O serogroups)^41,42^. Despite the high serological diversity among *Proteus* bacilli, the results of the presented research confirm the persisting for many years predominance of some serogroups reported earlier^10^ and reveal new prevalent O serotypes (especially O78). The presence of the representatives of different *Proteus* species among these serogroups suggests that the bacteria acquire these special O phenotypes as a result of adaptation in convergence processes. Thus, these O antigens may be in some ways attractive and beneficial to the expressing bacteria. It will be reasonable to investigate a clonal homology of one-species isolates belonging to the respective predominating O serogroups. Their diversity would confirm the special role of a particular O-antigen type in their survival and virulence.

The appointment of the most virulent O serotypes among the *Proteus* spp. bacilli would allow developing methods, which might enable rapid detection and eradication of such strains and the prophylaxis of recurrent complicated UTIs and CAUTIs.

## Supplementary Information

Supplementary Information 1.

Supplementary Information 2.

Supplementary Information 3.

Supplementary Information 4.

Supplementary Information 5.

Supplementary Information 6.

Supplementary Information 7.

Supplementary Information 8.

Supplementary Information 9.

Supplementary Information 10.

## Data Availability

The data and materials are available from the corresponding author on reasonable request.
