# Supplementary figures and images for: The prevailing O serogroups among the serologically differentiated clinical Proteus spp. strains in central Poland
Source: Sci Rep. 2021 Sep 23;11:18982. doi: 10.1038/s41598-021-98228-w (PMC8460819; doi:10.1038/s41598-021-98228-w)

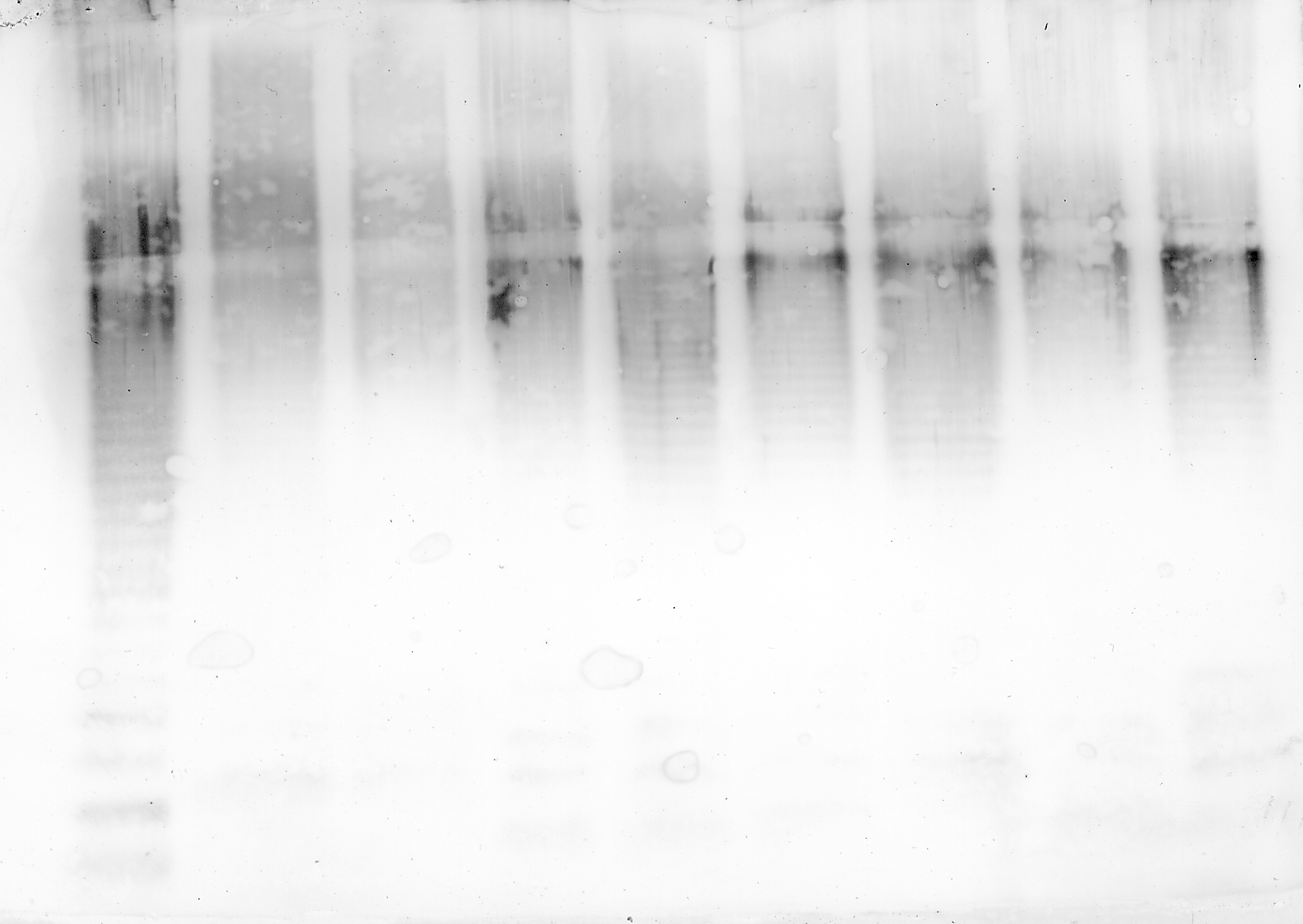

Supplement: Supplementary file 1 — Supplementary Information 1. [file 41598_2021_98228_MOESM1_ESM.tif]

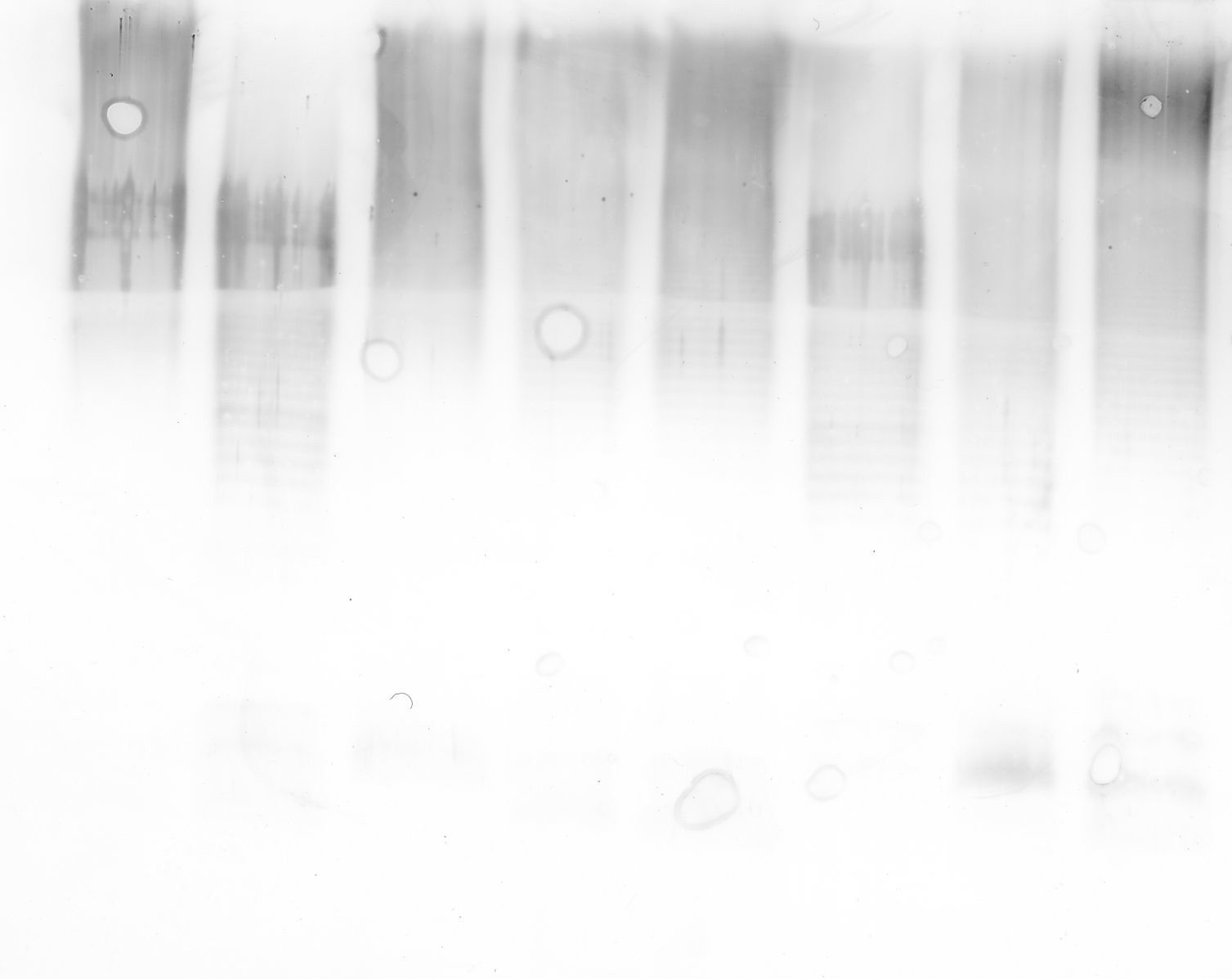

Supplement: Supplementary file 2 — Supplementary Information 2. [file 41598_2021_98228_MOESM2_ESM.tif]

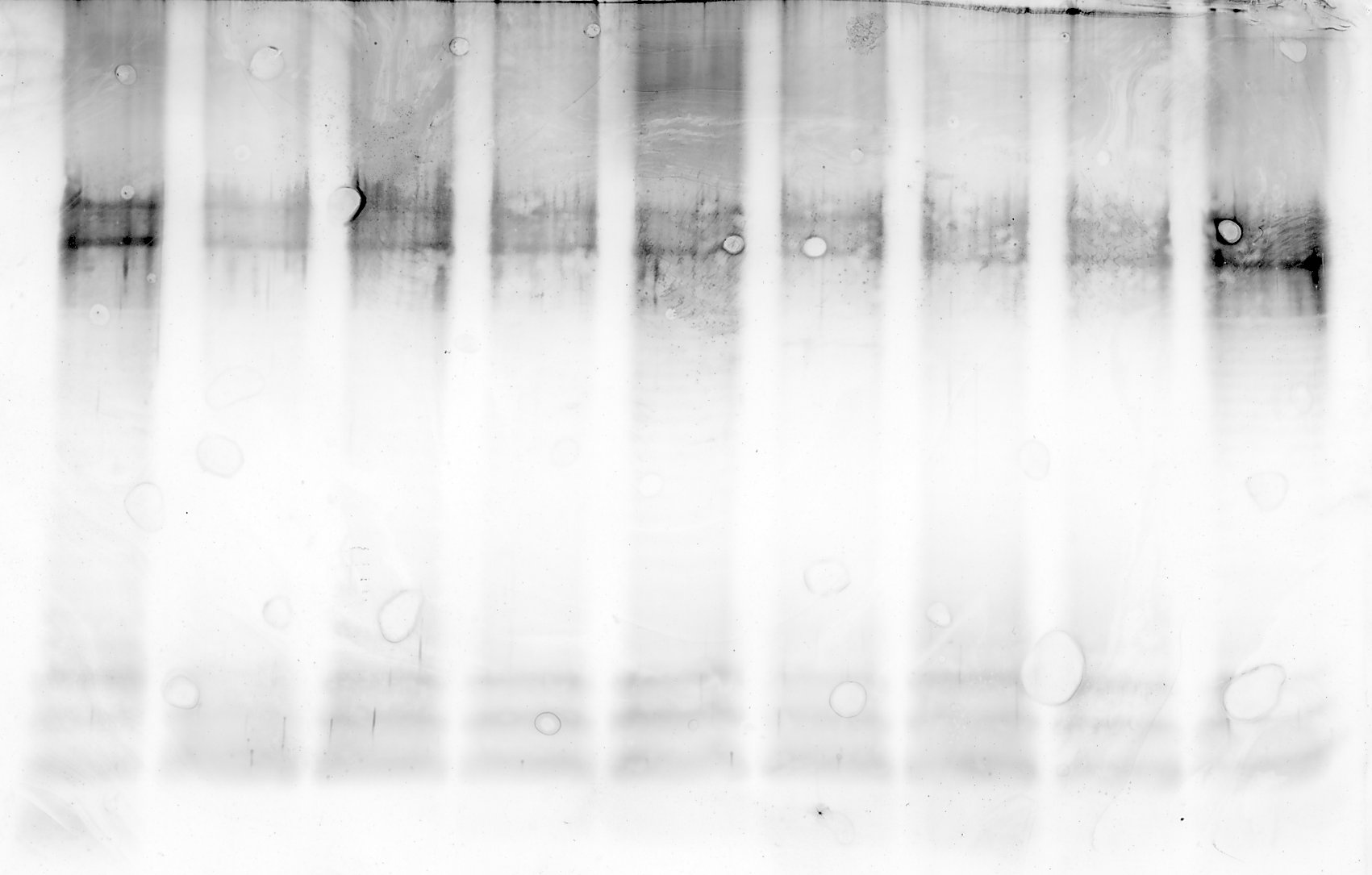

Supplement: Supplementary file 3 — Supplementary Information 3. [file 41598_2021_98228_MOESM3_ESM.tif]

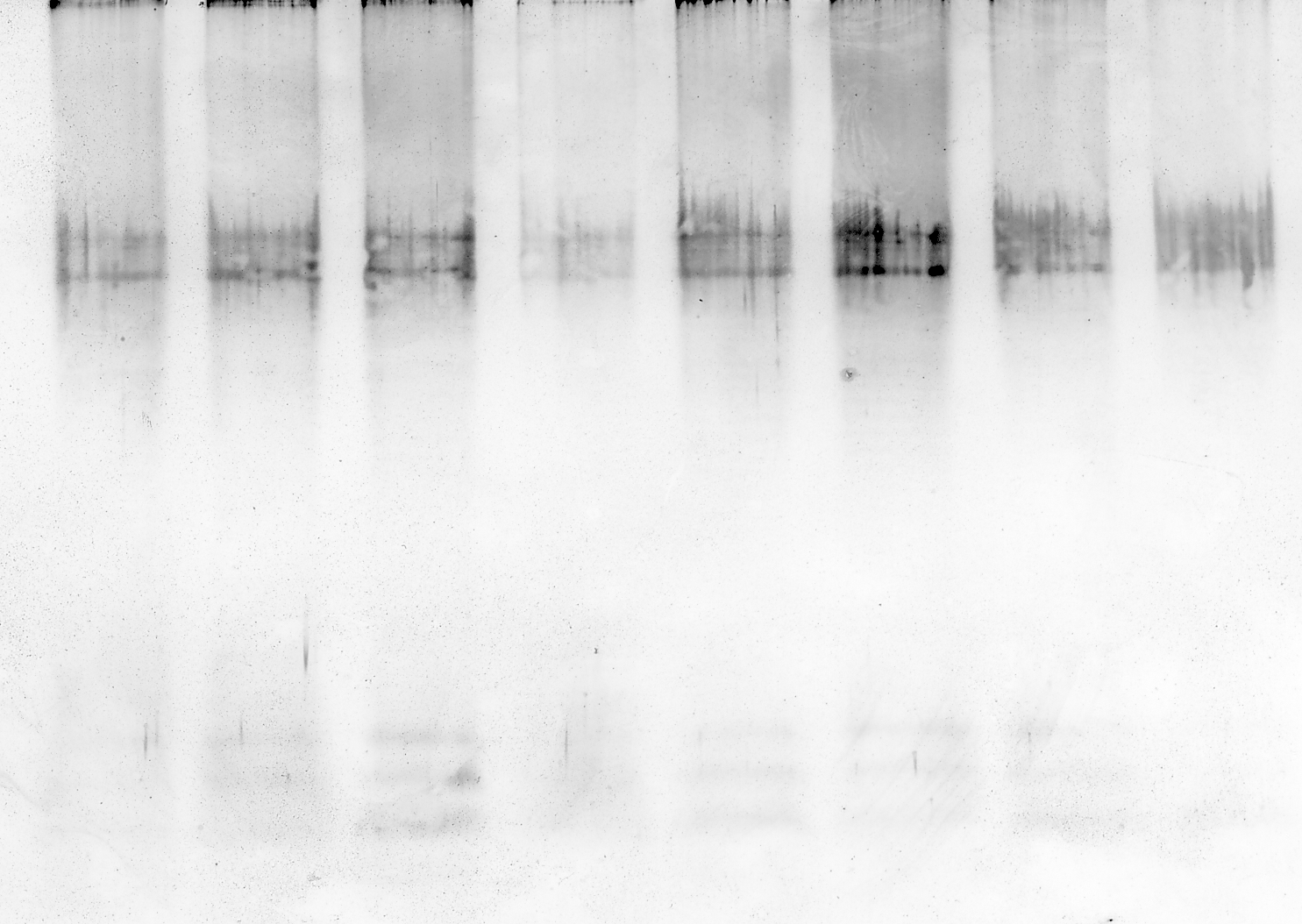

Supplement: Supplementary file 4 — Supplementary Information 4. [file 41598_2021_98228_MOESM4_ESM.tif]

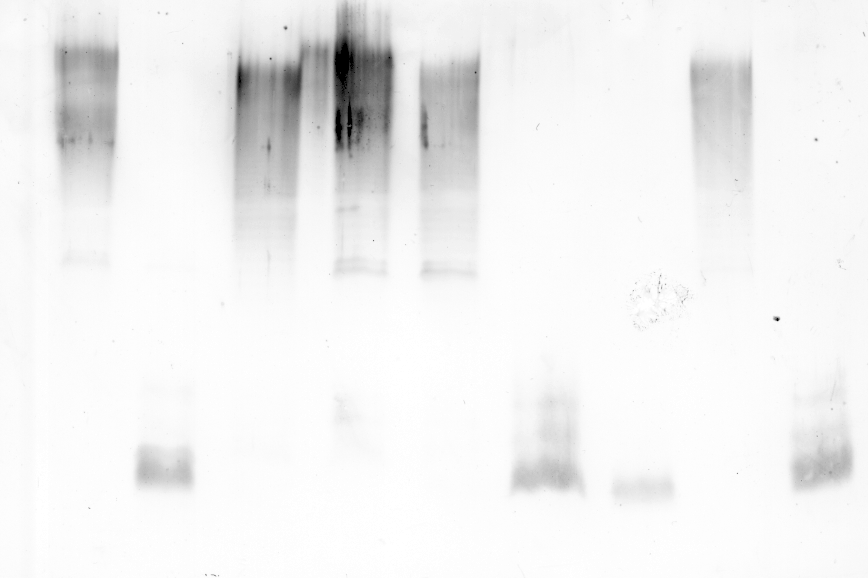

Supplement: Supplementary file 5 — Supplementary Information 5. [file 41598_2021_98228_MOESM5_ESM.tif]

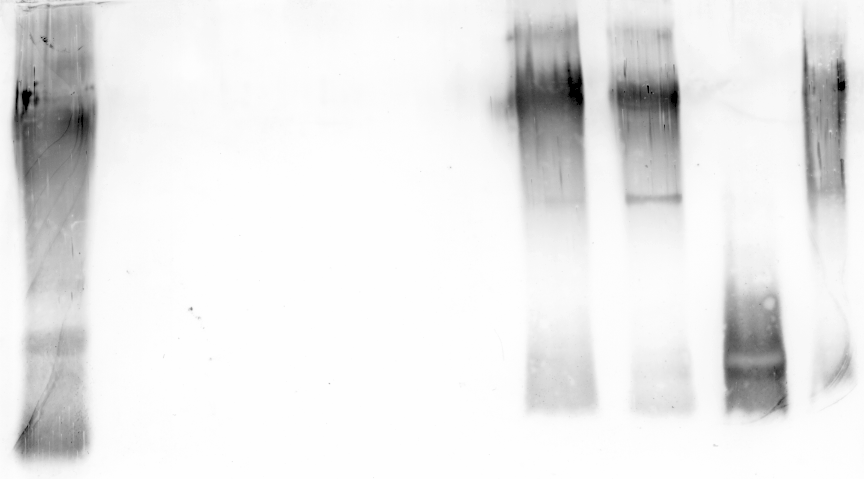

Supplement: Supplementary file 6 — Supplementary Information 6. [file 41598_2021_98228_MOESM6_ESM.tif]

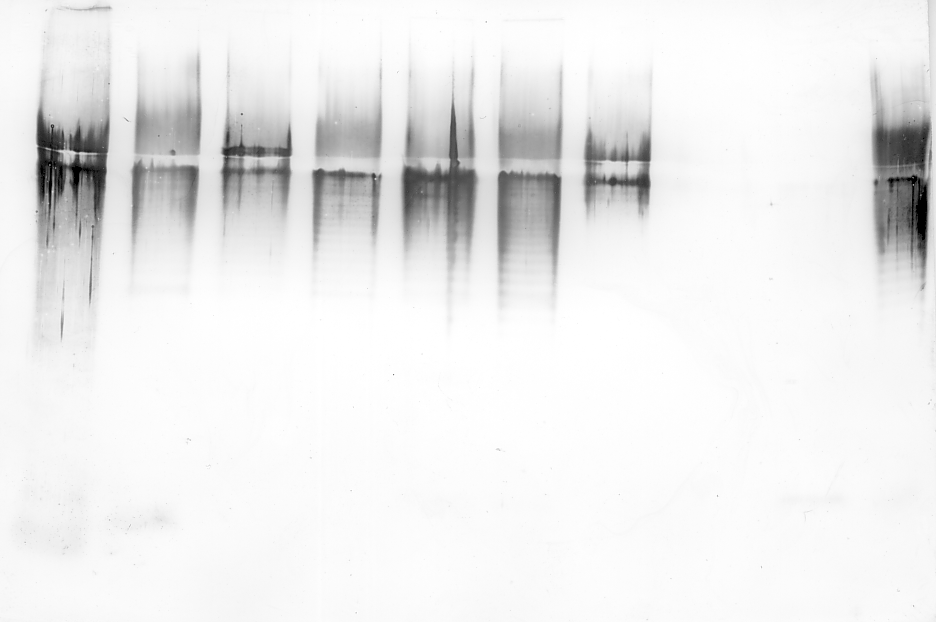

Supplement: Supplementary file 7 — Supplementary Information 7. [file 41598_2021_98228_MOESM7_ESM.tif]

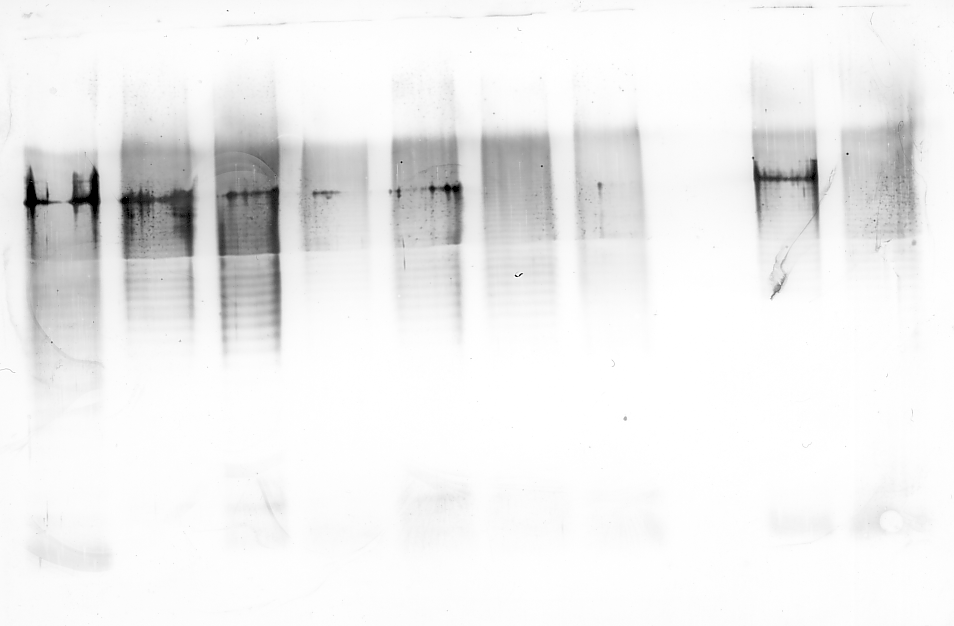

Supplement: Supplementary file 8 — Supplementary Information 8. [file 41598_2021_98228_MOESM8_ESM.tif]

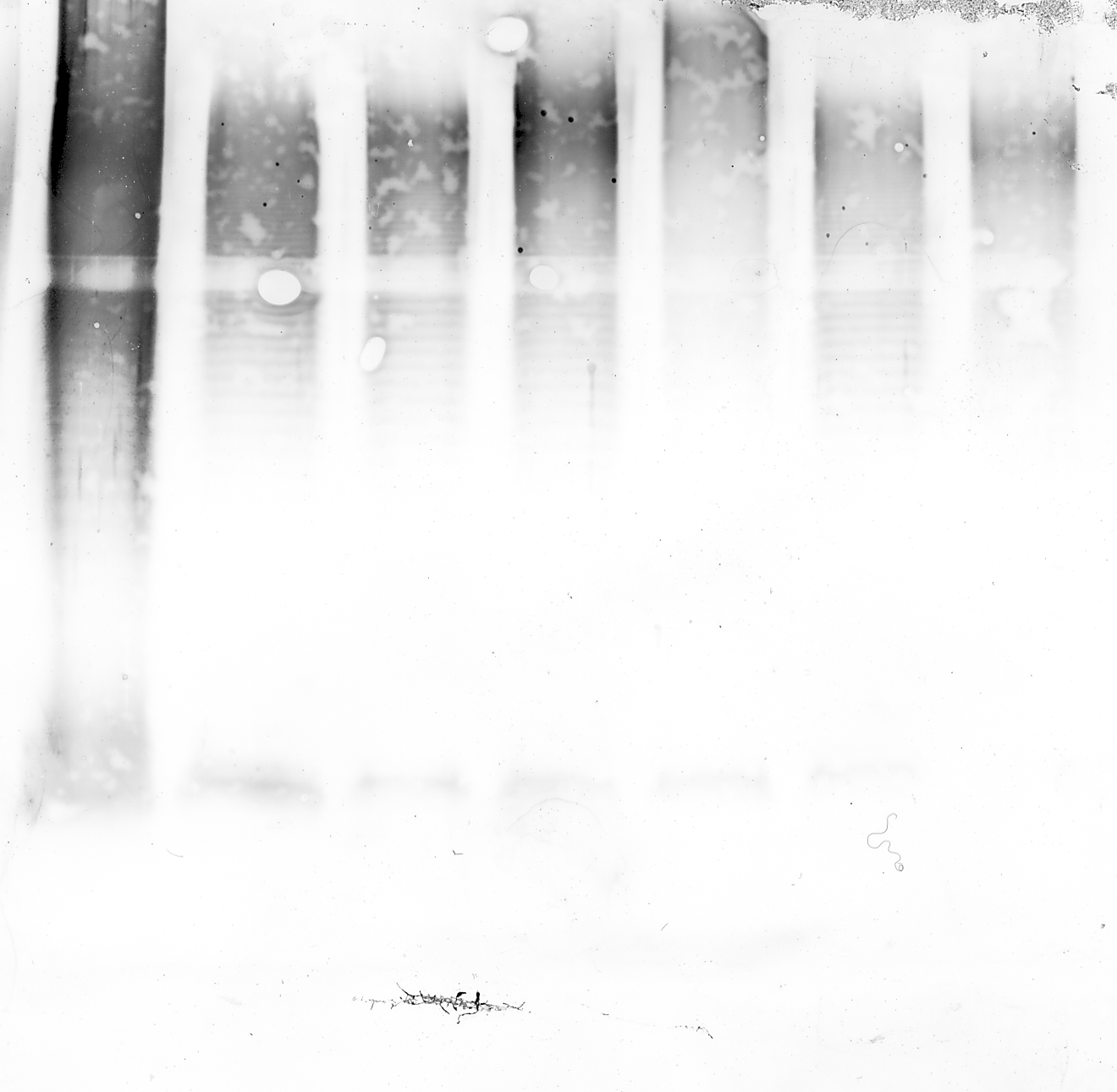

Supplement: Supplementary file 9 — Supplementary Information 9. [file 41598_2021_98228_MOESM9_ESM.tif]

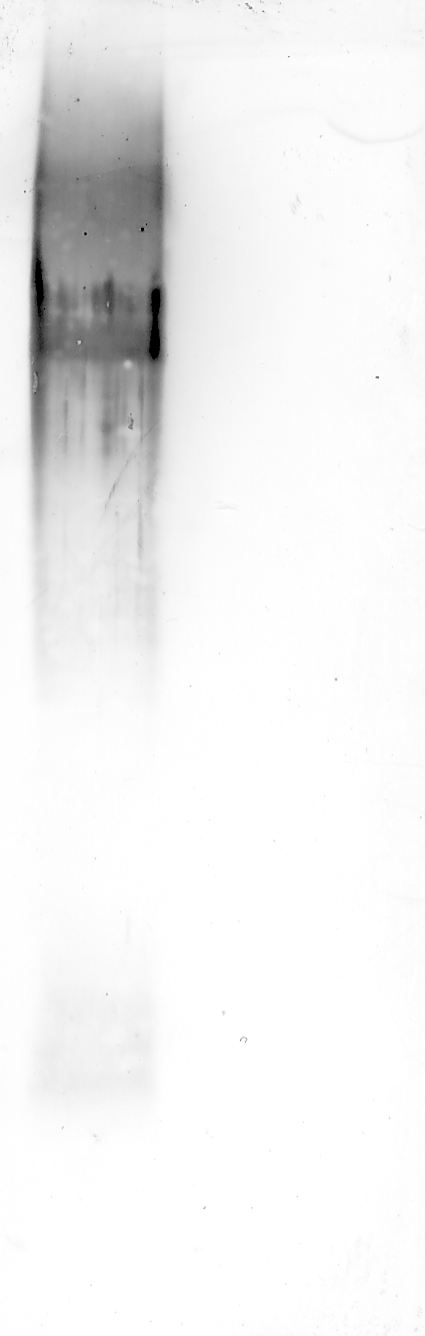

Supplement: Supplementary file 10 — Supplementary Information 10. [file 41598_2021_98228_MOESM10_ESM.tif]
